# Circulating *NPTX2* methylation as a non-invasive biomarker for prognosis and monitoring of metastatic pancreatic cancer

**DOI:** 10.1186/s13148-023-01535-4

**Published:** 2023-07-22

**Authors:** María Victoria García-Ortiz, Pablo Cano-Ramírez, Marta Toledano-Fonseca, María Teresa Cano, Elizabeth Inga-Saavedra, Rosa María Rodríguez-Alonso, Silvia Guil-Luna, María Auxiliadora Gómez-España, Antonio Rodríguez-Ariza, Enrique Aranda

**Affiliations:** 1 Maimónides Biomedical Research Institute of Córdoba (IMIBIC), Córdoba, Spain; 2 Andalusia-Roche Network Mixed Alliance in Precision Medical Oncology, Seville, Spain; 3 Cancer Network Biomedical Research Center (CIBERONC), Madrid, Spain; 4 Department of Anatomy and Comparative Pathology, University of Córdoba, Córdoba, Spain; 5 Medical Oncology Department, Reina Sofía University Hospital, Córdoba, Spain; 6 Department of Medicine, Faculty of Medicine, University of Córdoba, Córdoba, Spain

**Keywords:** *NPTX2*, Pancreatic ductal adenocarcinoma, Prognostic biomarker, Epigenetic biomarker, Liquid biopsy, DNA methylation, ddPCR, *RAS*, CA19-9

## Abstract

**Background:**

Pancreatic cancer is the most lethal cancer with a dismal prognosis mainly due to diagnosis at advanced stage and ineffective treatments. CA19-9 levels and computed tomography (CT) imaging are the main standard criteria for evaluating disease progression and treatment response. In this study we explored liquid biopsy-based epigenetic biomarkers for prognosis and monitoring disease in patients with metastatic pancreatic ductal adenocarcinoma (mPDAC).

**Methods:**

Plasma samples were collected from 44 mPDAC patients at the time of diagnosis, and in 15 of them, additional samples were obtained during follow-up of the disease. After cell-free DNA (cfDNA), isolation circulating levels of methylated *NPTX2*, *SPARC*, *BMP3*, *SFRP1* and *TFPI2* genes were measured using digital droplet PCR (ddPCR). BEAMing technique was performed for quantitation of *RAS* mutations in cfDNA, and CA19-9 was measured using standard techniques.

**Results:**

*NPTX2* was the most highly and frequently methylated gene in cfDNA samples from mPDAC patients. Higher circulating *NPTX2* methylation levels at diagnosis were associated with poor prognosis and efficiently stratified patients for prediction of overall survival (6.06% cut-off, *p* = 0.0067). Dynamics of circulating *NPTX2* methylation levels correlated with disease progression and response to therapy and predicted better than CA19-9 the evolution of disease in mPDAC patients. Remarkably, in many cases the disease progression detected by CT scan was anticipated by an increase in circulating *NPTX2* methylation levels.

**Conclusions:**

Our study supports circulating *NPTX2* methylation levels as a promising liquid biopsy-based clinical tool for non-invasive prognosis, monitoring disease evolution and response to treatment in mPDAC patients.

**Supplementary Information:**

The online version contains supplementary material available at 10.1186/s13148-023-01535-4.

## Background

Pancreatic cancer is the 3rd tumor with the highest mortality rate in developed countries and the tumor with the lowest (9%) 5-year survival [1, 2]. In Europe, this disease causes around 95,000 deaths every year [3] and its incidence has been increasing in recent years, with age 65 to 70 years at the time of diagnosis. Pancreatic ductal adenocarcinoma (PDAC) represents more than 80% of all pancreatic neoplasms [4], being a tumor that is difficult to diagnose during the initial stages, very aggressive, with rapid progression and a very poor prognosis. The absence of specific symptoms in the early stages of the disease and the lack of effective diagnostic methods are the main reasons for this dismal prognosis. The consequence is that more than 75% of patients are diagnosed with locally advanced or metastatic disease and only 15–20% are operable at the time of diagnosis. Despite surgical resection, patients relapse early, with a median survival of only 10–20 months [5, 6]. In patients with metastatic disease, the median overall survival from diagnosis is 4.6 months [7]. Therefore, effective biomarkers are needed for prognosis at the time of diagnosis, as well as for the follow-up of patients to predict early relapse after surgical resection or therapeutic failure in patients with advanced disease.

To date, the carbohydrate antigen 19-9 (CA19-9) is the only blood-based biomarker routinely used to make clinical decisions in pancreatic cancer, with a relatively low sensitivity (79%) and specificity (82%) [8]. There is a relationship between CA19-9 levels and survival in patients with metastatic PDAC [9], but in clinical practice there is no consensus on the interpretation of changes in CA19-9 levels during the course of the disease [10].

Compared to tumor biopsies, analysis of cell-free DNA (cfDNA) in liquid biopsies provides a better description of the complete landscape of a tumor. Additionally, cfDNA offers the benefit of sequential sampling, allowing dynamic evaluation of changes in cfDNA concentration, identification of acquired resistance-conferring mutations, and monitoring of clonal evolution [11].

In recent years, there has been a significant increase in the number of studies aimed at identifying DNA methylation markers in cancer [12]. The process of DNA methylation involves the addition or removal of a methyl group at the C5 position of cytosine within CpG dinucleotides, primarily located in specific genomic regions called CpG islands. In normal cells, accurate DNA methylation patterns ensure the precise regulation of gene expression and maintaining stable gene silencing. Thus, aberrant methylation of promoter regions of certain tumor suppressor genes has been described to lead to gene silencing, contributing to the onset and progression of cancer [13, 14]. Besides, DNA methylation offers a significant advantage over genetic alterations due to its reversibility, making it an exceptionally valuable biomarker with highly relevant therapeutic potential [15]. Thus, promoter methylation of several genes has been proposed as promising non-invasive prognostic markers in different types of cancer [16, 17]. However, there are very few studies on the prognostic value of hypermethylated cfDNA in PDAC. After conducting a thorough analysis of published studies on cfDNA methylation in pancreatic cancer, as well as a comparative methylation analysis using the TCGA database, we have identified a set of consistently reported hypermethylated genes: *BMP3*, *NPTX2*, *SFRP1*, *SPARC*, and *TFPI2* [18–20]. These genes not only serve as potential biomarkers for pancreatic tumor disease but have also been proposed as promising early blood-based diagnostic tool.

In the present study, we aimed to investigate the potential of circulating epigenetic markers for prognosis and monitoring disease in patients with metastatic pancreatic cancer. Hence, we have employed the droplet digital PCR (ddPCR), as a robust, sensitive and specific technology, to examine the cfDNA methylation levels of *BMP3*, *NPTX2*, *SFRP1*, *SPARC* and *TFPI2* genes. This approach allowed us to investigate their potential as prognostic and management tools for patients with metastatic pancreatic cancer.

## Methods

### Patients and samples

A cohort of 44 patients was prospectively included in this study at the Reina Sofia University Hospital (HURS, Cordoba, Spain) between May 2017 and February 2022. The inclusion criteria were patients over 18 years with histologically confirmed metastatic pancreatic ductal adenocarcinoma, without chemotherapy or radiotherapy before enrollment and with signed informed consent. The study was conducted in accordance with the World Medical Association Declaration of Helsinki, and the protocol was approved by the Ethics Committee of Córdoba (PANCREAS-BIOPSIA-LIQ protocol, approved on April 26, 2017, Act no263, ref, 3490). The baseline characteristics of the patients included in the study are summarized in Table 1.

**Table 1:** Clinicopathological characteristics of patients

Patient characteristics	Number of patients (*n* = 44)	Percentage
Age		
≤ 66 years	24	54.5
> 66 years	20	45.5
Sex		
Male	27	61.4
Female	17	38.6
Stage		
IV	44	100
ECOG		
0	12	27.2
1	23	52.3
2	8	18.2
3	1	2.3
Primary tumor location		
Body	19	43.2
Tail	12	27.3
Head	13	29.5
First-line treatment		
Gemcitabine/ ± nab-paclitaxel	37	84.1
FOLFIRINOX	4	9.1
No treatment	3	6.8
Number of metastatic lesions		
One	24	54.6
More than one	20	45.4
Metastatic lesions location		
Hepatic	36	81.8
Non-hepatic	8	18.2
Tissue Biopsy *RAS* status^a^		
*RAS* mutated	20	69
*RAS* wild-type	9	31
Liquid Biopsy *RAS* status		
*RAS* mutated	35	79.5
*RAS* wild-type	9	20.5

^a^For the analysis of *RAS* mutational status, primary tumor tissue was available in 65.9% (29/44) of patients

A total of 95 plasma samples were analyzed, comprising 44 basal samples at diagnosis and 51 samples from the follow-up (monitoring) of 15 patients. Monitoring samples were obtained to coincide with the evaluation of disease progression by CT scan imaging, until disease progression or death of the patient. Plasma was obtained from 10 mL of blood collected in Streck cell-free DNA BCT™ tubes. Blood samples were centrifuged at 1600 × *g*, 10 min at room temperature (RT) to separate plasma, that was then centrifuged at 6000 × *g*, 10 min at RT to remove possible cellular debris. The plasma samples were then aliquoted into cryotubes and stored at − 80 °C until use. CA19-9 levels were measured in the Clinical Laboratory Department of our hospital using a standard radioimmunoassay test.

### Plasma cfDNA isolation

cfDNA was extracted from 3 ml of plasma using the QIAamp Circulating Nucleic Acid Kit and the QIAvac 24 Plus vacuum system (Qiagen), and quantified using the Quantus fluorometer (Promega).

### Circulating *RAS* mutation analysis

Analysis of *RAS* mutations in cfDNA and determination of mutant allele fraction (MAF) were performed using the OncoBEAM™ RAS assay (Sysmex Inostics GmbH), as previously described [21].

### Analysis of cfDNA methylation

Bisulfite conversion of isolated cfDNA was performed using a maximum sample volume of 25 μl with the EZ DNA Methylation-Lightning™ kit (Zymo Research), according to the manufacturer’s instructions. For each bisulfite treatment, 3 control samples (Zymo Research) were incorporated to confirm proper realization of the modification treatment (Human HCT116 DKO Methylated DNA, Human HCT116 DKO Non-Methylated DNA and a 50% mixture of both).

### Droplet digital PCR (ddPCR)

Before ddPCR, and when a preamplification step was performed, a reaction mixture (10 μl) was prepared with bisulfite converted cfDNA (1 μL), 5 μL of 2 × ddPCR Supermix (without dUTP; Bio-Rad), 900 nM of forward and reverse primers (in case of two reverse primers, M and U, 450 nM of each were added). PCR protocol was as follows: 95 °C for 10 min, 10 cycles of 94 °C for 30 s and 60 °C for 1 min and a final step at 98 °C for 10 min. Finally, all preamplified PCR products were diluted 1:10.ddPCR was performed with the QX200 Droplet Digital PCR System (Bio-Rad). In a final volume of 20 μl, 2 μl of template DNA —bisulfite-treated cfDNA or diluted preamplified PCR product— was mixed with ddPCR Supermix, primers (900 nM of each primer, forward and reverse (in case of two reverse primers, M and U, 450 nM of each were added)) and corresponding probes (FAM or SUN probes 250 nM, synthesized by IDT, Integrated DNA Technologies, Inc.). To reduce background noise and increase assay sensitivity, all probes were quenched with double quenchers (IDT), a 30 Iowa Black dark quencher (IABkFQ) combined with an internal ZEN quencher. The sequence of primers and probes is specified in Additional file 1: Table S1. ddPCR conditions were as follows: 95 °C for 10 min, 40 cycles of 94 °C for 30 s, 60 °C for 1 min and a final step at 98 °C for 10 min. PCR products were analyzed in the droplet reader, which determined the total number of droplets formed, as well as the number of positive and negative droplets for each fluorophore.

The results were analyzed with the program QuantaSoft™ Analysis Pro 1.0.596 (Bio-Rad), which uses a Poisson distribution to calculate methylation ratios or relative abundance. When *β-Actin* was used as reference gene for normalization, the methylation ratio was defined as number of methylated copies from the target gene (FAM)/ number of positive copies from the *ACTB* gene (SUN). When unmethylated molecules of *NPTX2* gene were used for normalizing, the relative abundance of methylation was defined as [number of methylated copies (FAM) of *NPTX2* gene/(number of methylated copies of *NPTX2* gene (FAM) + unmethylated copies (SUN) of *NPTX2* gene)]*100.

### Statistical analysis

Graphs and data analysis were performed with GraphPad Prism 9.3.1 Software. Optimal cut-off values were selected using values at diagnosis through the median value (for cfDNA concentration, CA19-9 levels and *RAS* MAF) or by receiver operating characteristics (ROC) curve (for *NPTX2* methylation). Statistical significance was determined using the non-parametric Mann–Whitney U test. Association and/or correlation studies were performed using Spearman's correlation coefficient. Overall survival (OS) was calculated from diagnosis to death from any cause. Progression-free survival (PFS) was calculated from the start date of therapy until disease progression or death. The survival rates were estimated using the Kaplan–Meier method, and the log-rank test was used to identify prognostic variables. A *p* value < 0.05 was considered to indicate a statistically significant difference.

## Results

### Analysis of patient clinicopathological characteristics

A total of 95 plasma samples were obtained from 44 patients diagnosed with distant metastases from PDAC between 2017 and 2022. Peripheral blood samples at the time of diagnosis were obtained from all patients before receiving any treatment. Blood samples were also obtained during follow-up of patients and at the same time as CT imaging of disease evolution.

The clinicopathological characteristics of patients are shown in Table 1. Patients were 27 males and 17 females, with a median age of 66 years (range 54–84 years). Primary tumor was located in the body (43.2%), head (29.5%) or tail (27.3%) of the pancreas, and the main distant metastatic location (81.8%) was the liver. Most patients (79.5%) had a good baseline ECOG (ECOG 0–1) and 81.4% received first-line gemcitabine-based regimens. *RAS* mutations were detected in 69% and 79.5% of tumor tissue and basal plasma samples, respectively. The overall concordance between tissue and plasma *RAS* analysis was 75.9%.

There was disease progression in 100% of the patients and all had died at the time of preparing this manuscript.

When patients were stratified according to sex, age, ECOG or primary tumor location, no differences in overall survival and progression-free survival were found (Table 2). Non-hepatic metastatic location was related with better OS (*p* = 0.029) and better PFS (*p* = 0.004), whereas there was no significant association between number of metastasis and OS or PFS.

**Table 2:** Overall survival (OS) and progression-free survival analysis (PFS)

Variables	OS		PFS	
	HR (95% CI)	*p*	HR (95% CI)	*p*
Age				
≤ 66 years	0.8249	0.512	0.7509	0.340
> 66 years^R^	(0.456–1.489)		(0.409–1.376)	
Sex				
Male^R^	1.715	0.060	1.740	0.059
Female	(0.949–3.099)		(0.948–3.193)	
ECOG				
0^R^				
1	0.790 (0.402–1.556)	0.492	0.6893 (0.348–1.365)	0.288
2–3	0.619 (0.247–1.554)	0.167	0.43 (0.1496–1.236)	0.047
Primary tumor location				
Head^R^	1.16	0.624	1.295	0.44
Body/tail	(0.596–2.275)		(0.6353–2.640)	
Number of metastasis				
1	1.19	0.555	1.301	0.383
≥ 2^R^	(0.652–2.169)		(0.703–2.405)	
Metastatic location				
Hepatic^R^	2.193	0.029	2.678	0.004
Non-hepatic	(1.163–4.133)		(1.431–5.012)	
*RAS* status in plasma				
MUT^R^	2.605	0.003	2.850	0.0007
WT	(1.425–4.760)		(1.549–5.243)	
MAF				
> 2.01%^R^	1.591	0.107	1.813	0.035
< 2.01%	(0.867–2.920)		(0.963–3.409)	
CA19-9				
> 4515 U/mL^R^	1.517	0.145	1.554	0.141
< 4515 U/mL	(0.821–2.803)		(0.827–2.920)	
cfDNA concentration				
> 31.8 ng/mL^R^	1.858	0.032	1.853	0.034
< 31.8 ng/mL	(0.999–3.451)		(0.973–3.526)	
*NPTX2* methylation				
> 6.06%^R^	2.190	0.006	1.844	0.032
< 6.06%	(1.186–4.044)		(0.989–3.437)	

^R^Reference category for analysis

### Methylation status of *BMP3*, *NPTX2*, *SPARC*, *SFRP1* and *TFPI2* in cfDNA from metastatic PDAC patients

Initially, analysis of pancreatic adenocarcinoma TCGA data confirmed that the methylation levels of *BMP3*, *NPTX2*, *SPARC*, *SFRP1* and *TFPI2* genes were significantly higher in tumor compared with normal tissue, reinforcing their potential as useful biomarkers (Additional file 2: Fig. S1).

Therefore, and due to the great precision and technical simplicity of ddPCR [22], we decided to perform the cfDNA methylation status analysis of these five selected genes using this approach.

**Fig. 1:**
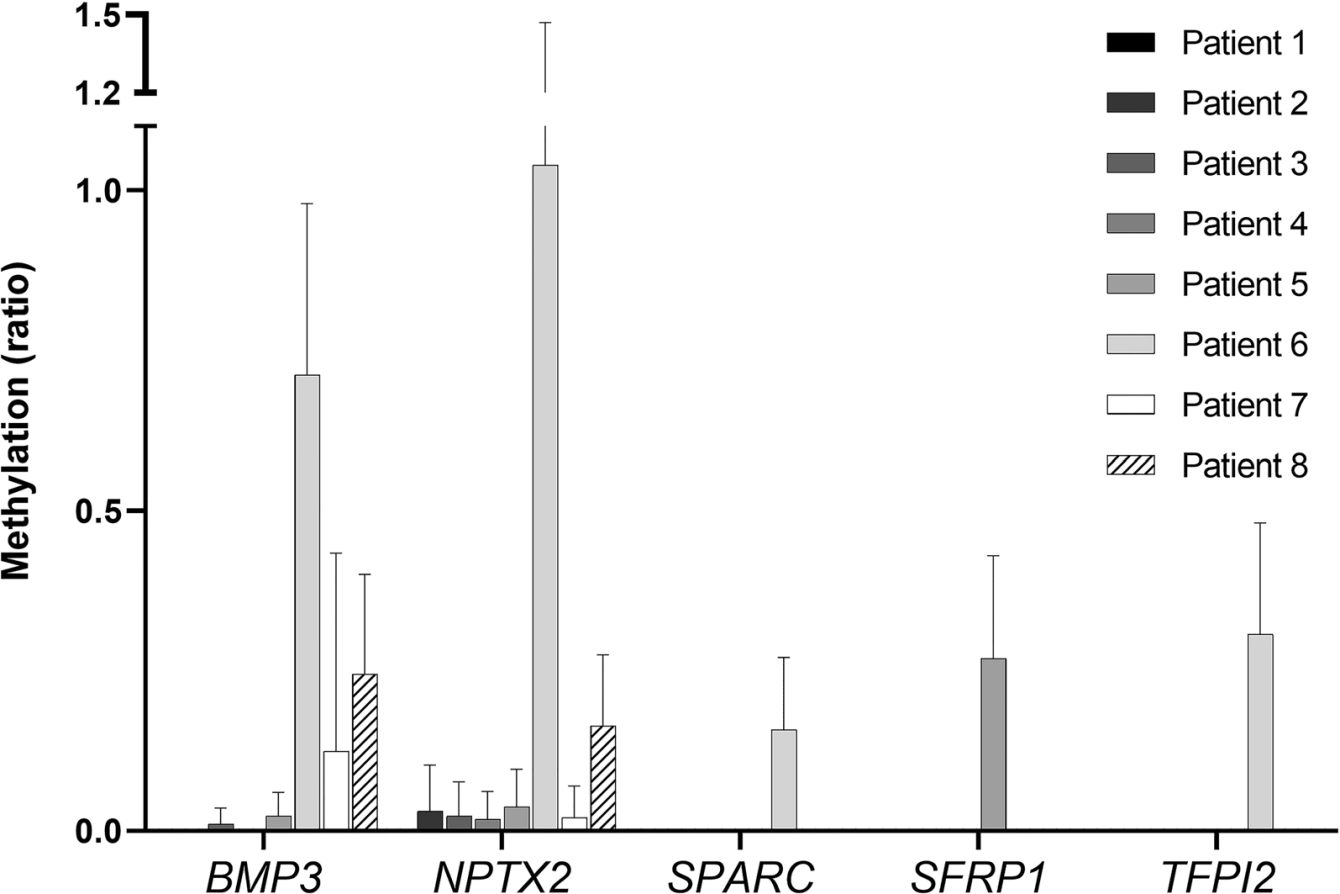
Methylation levels of *BMP3*, *NPTX2*, *SPARC*, *SFRP1*, and *TFPI2* in cfDNA from mPDAC patients. The graph shows the methylation ratio of each gene in eight patients at the time of diagnosis. The methylation ratio is defined as the number of methylated copies of the target gene/number positive copies of the *ACTB* gene. Error bars indicate Poisson’s error (95% CI). The ddPCR analysis of cfDNA from mPDAC patients revealed *NPTX2* as the most frequently methylated gene

Firstly, analysis was performed in basal and monitoring samples from eight metastatic PDAC (mPDAC) patients using *β-Actin* as reference gene for normalization of methylation levels. Results indicated that *NPTX2* was the most frequently methylated gene in cfDNA samples (Fig. 1), being methylated in 87.5% of baseline samples, and in 66.7% of monitoring samples (Table 3). The methylation frequency of the rest of genes was much lower, except for *BMP3*, which was methylated in 62.5% of baseline samples, but only in 16.6% of monitoring samples. Finally, the lowest methylation levels were found for *SPARC*, *SFRP1* and *TFPI2* genes. In this regard, it is important to note that TCGA data also pointed to *NPTX2* as the most differentially methylated in PDAC (Additional file 2: Fig. S1).

**Table 3:** Monitoring of *NPTX2* methylation frequencies

Methylation monitoring	*n*	*BMP3*^*m*^	*NPTX2*^*m*^	*SPARC*^*m*^	*SFRP1*^*m*^	*TFPI2*^*m*^
To diagnosis	8	5 (62.5%)	7 (87.5%)	1 (12.5%)	1 (12.5%)	1 (12.5%)
After treatment	24	4 (16.67%)	16 (66.67%)	1 (4.17%)	3 (12.5%)	1 (4.17%)

Therefore, we decided to focus on *NPTX2* methylation in cfDNA from mPDAC patients. On the other hand, pancreatic tumors are characterized by lower levels of DNA shed into circulation in comparison with other tumor types [23]. Therefore, to improve the determination of *NPTX2* methylation levels in cfDNA by ddPCR, a previous amplification step of analyzed fragments was introduced and the number of unmethylated molecules of *NPTX2* gene was used as a normalizing factor. Representative ddPCR results obtained in two patients (14 and 15) using this approach are shown in Additional file 3: Fig. S2.

The Poisson model is a correction factor that considers the inhomogeneous distribution of DNA molecules in each well of a ddPCR plate. Therefore, we compared Poisson's error values (95% CI) from ddPCR analyses of *NPTX2* methylation with and without preamplification in the baseline and monitoring samples (66) from 15 patients (Additional file 1: Table S2). The Poisson’s error obtained using preamplification was lower than that without preamplification in 97.8% of the samples (mean ± 3.10), suggesting that a preamplification step before ddPCR provides higher sensitivity and reliability in samples with limited amounts of DNA. In addition, *NPTX2* methylation controls were performed using cfDNA from healthy donors as well as fully methylated and unmethylated commercial DNA samples. *NPTX2* methylation was not detected in cfDNA from healthy donors, whereas all commercial controls showed the expected levels of *NPTX2* methylation (Additional file 4: Fig. S3). This was also observed for all the other examined genes (Additional file 5: Fig. S4).

### Association of circulating *NPTX2* methylation with other circulating tumor biomarkers in metastatic PDAC

The potential association between circulating *NPTX2* methylation levels and other circulating tumor biomarkers, such as *RAS* mutational status and *RAS* mutant allele fraction in cfDNA, CA19-9 levels or cfDNA concentration, was determined in all 95 liquid biopsy samples. Cut-off values for each biomarker, which were calculated from mean values at diagnosis, were 31.8 ng/mL for cfDNA concentration, 4515 U/mL for CA19-9 and 2.01% for *RAS* mutant allele fraction.

**Fig. 2:**
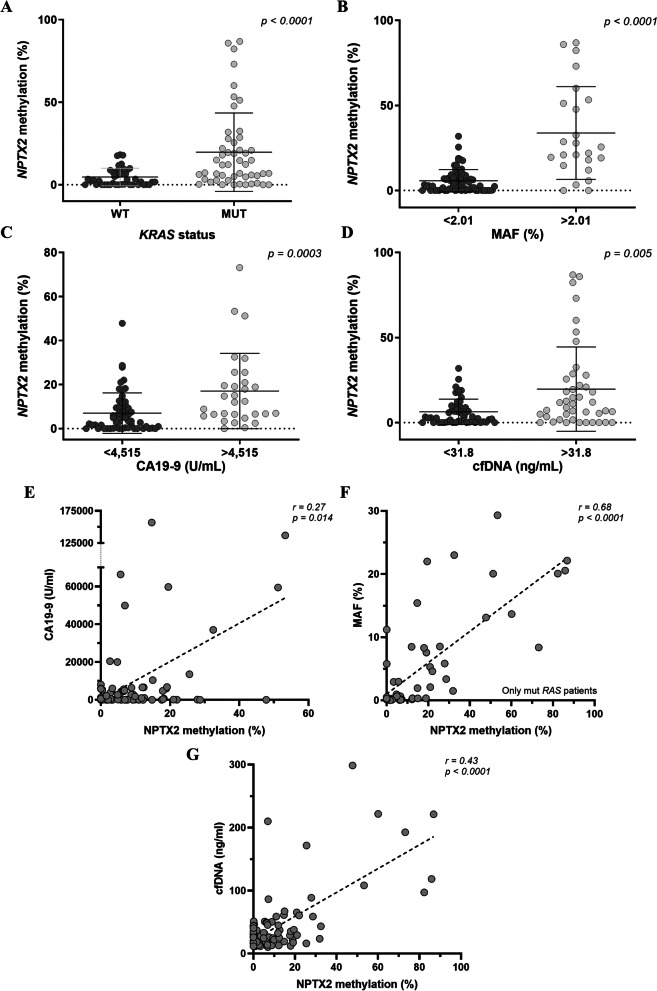
Association of circulating *NPTX2* methylation with other circulating tumor biomarkers in mPDAC. **A**–**D** Plasma *NPTX2* methylation levels according to their *RAS* status (**A**), *RAS* mutant allele fraction (MAF) (**B**), CA19-9 (**C**) and cfDNA concentration (**D**) cut-off values, which were set at 2.01% for *RAS* MAF, 4515 U/mL for CA19-9 and 31.8 ng/mL for cfDNA concentration. Data are shown as mean ± SD for each group. *NPTX2* was significantly more methylated in those samples with mutated *RAS*, with a higher MAF of *RAS*, with higher CA19-9 values and with higher cfDNA concentration. **E**–**G** Correlation analysis of circulating *NPTX2* methylation with CA19-9 levels (**E**), MAF value (**F**) and cfDNA concentration (**G**)

The *NPTX2* methylation levels were significantly higher (*p* < 0.0001) in those plasma samples with *RAS* mutated cfDNA (Fig. 2A). Accordingly, higher *NPTX2* methylation levels were also significantly associated with a higher mutant allele fraction of *RAS* (23.80% vs. 3.47%, *p* < 0.0001) (Fig. 2B). *NPTX2* was also significantly more methylated in those samples with CA19-9 values above 4515 U/mL (12.20% vs. 3.43%, *p* = 0.0003) (Fig. 2C). Besides, those samples with higher cfDNA levels displayed significantly higher *NPTX2* methylation levels (11.0% vs. 3.3%, *p* = 0.005) (Fig. 2D).

Moreover, a significant correlation was observed between circulating *NPTX2* methylation and CA19-9 levels (*r* = 0.27, *p* = 0.014), circulating *RAS* MAF (*r* = 0.68, *p* < 0.0001) or cfDNA concentration (*r* = 0.43, *p* < 0.0001) (Fig. 2E–G).

### Circulating *NPTX2* methylation status is a prognostic biomarker in mPDAC

**Fig. 3:**
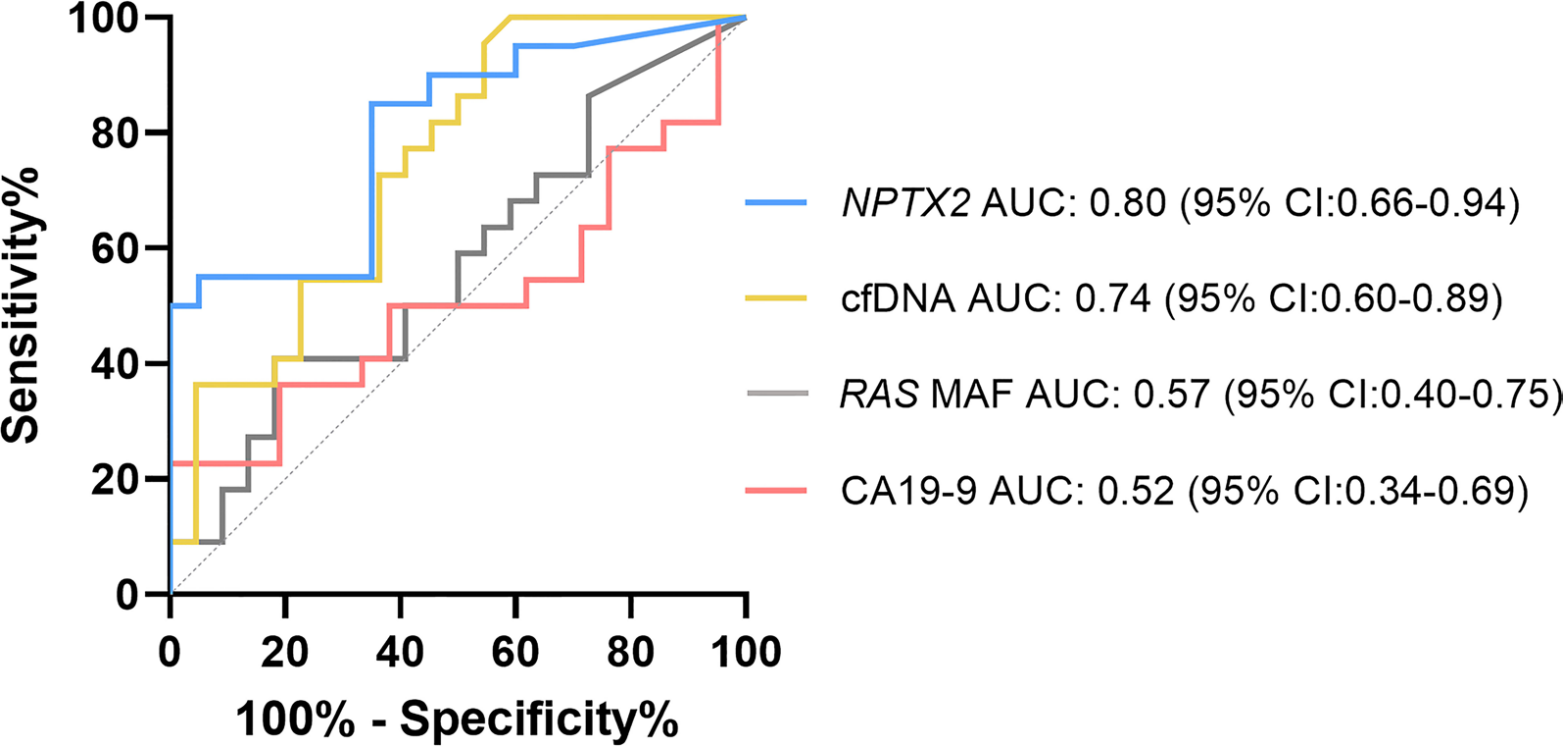
Prognostic performance of circulating *NPTX2* methylation in comparison with other circulating tumor biomarkers in mPDAC patients. Receiving operating characteristic (ROC) curves of plasma *RAS* MAF, CA19-9, cfDNA concentration and *NPTX2* methylation in the prognosis of mPDAC. The higher prognostic value was observed for *NPTX2* methylation levels (AUC 0.80, 95% CI 0.66–0.84)

Next, we evaluated the prognostic performance of circulating *NPTX2* methylation in comparison with the other circulating biomarkers analyzed. First, receiver operating characteristic (ROC) curves were constructed for survival prediction at the median survival (239 days) of the patients. As shown in Fig. 3, *NPTX2* methylation (6.06% cut-off) was the best predictive circulating biomarker, with 85% sensitivity, 65% specificity, and an area under the curve (AUC) of 0.80 (95% CI 0.66–0.94).

**Fig. 4:**
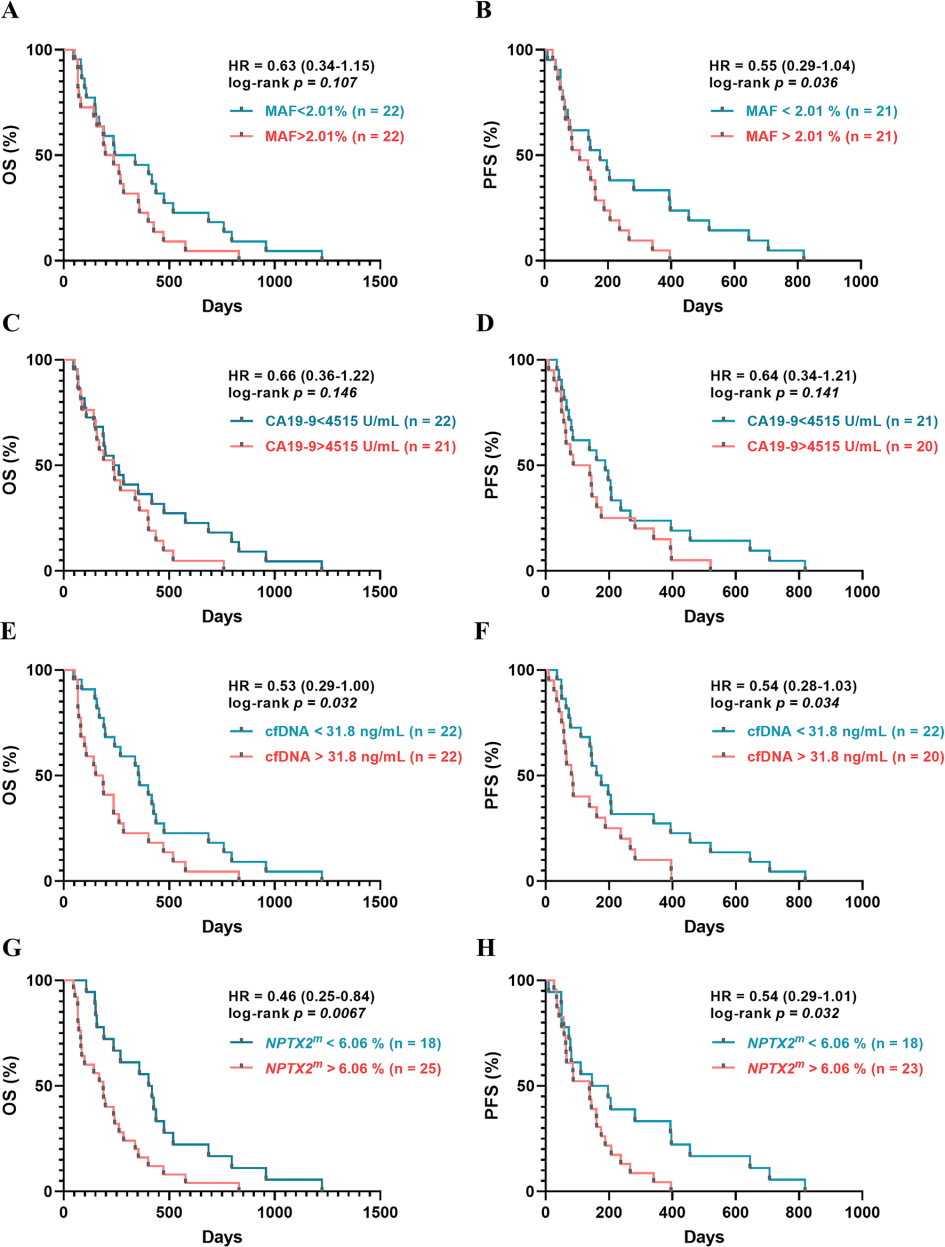
Circulating *NPTX2* methylation status as prognostic biomarker in mPDAC patients. **A** Overall survival (OS) according to plasma *RAS* mutant allele fraction (MAF). **B** Progression-free survival (PFS) according to plasma *RAS* MAF. **C** OS according to CA19-9 level. **D** PFS according to CA19-9 level. **E** OS according to cfDNA concentration. **F** PFS according to cfDNA concentration. **G** OS according to *NPTX2* methylation. **H** PFS according to *NPTX2* methylation

To further explore the prognostic power of circulating *NPTX2* methylation levels, analysis of overall survival and progression-free survival based on Kaplan–Meier curves were performed with the median basal levels of each circulating biomarker and the cut-off established by the ROC curve for the *NPTX2* methylation (Table 2 and Fig. 4). As we reported previously, higher cfDNA concentrations and higher *RAS* mutational load in cfDNA were associated with poor prognosis in mPDAC patients [21]. Remarkably, *NPTX2* methylation also significantly stratified mPDAC patients. Specifically, *NPTX2* methylation (6.06% cut-off) distinguished between low-risk (410 OS days) and high-risk (187 OS days) groups (*p* = 0.0067) (Fig. 4G).

### Circulating *NPTX2* methylation status for monitoring mPDAC patients

**Fig. 5:**
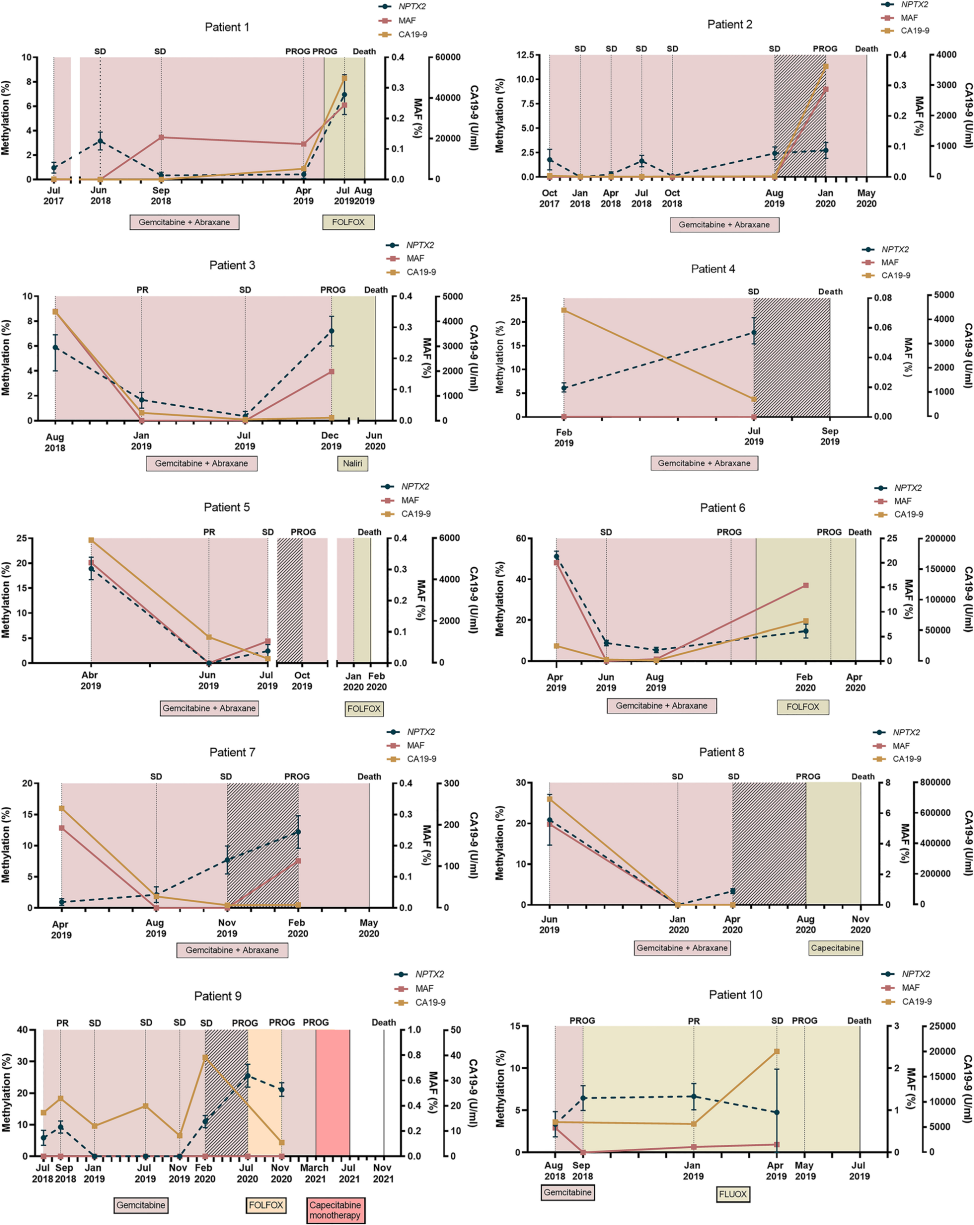

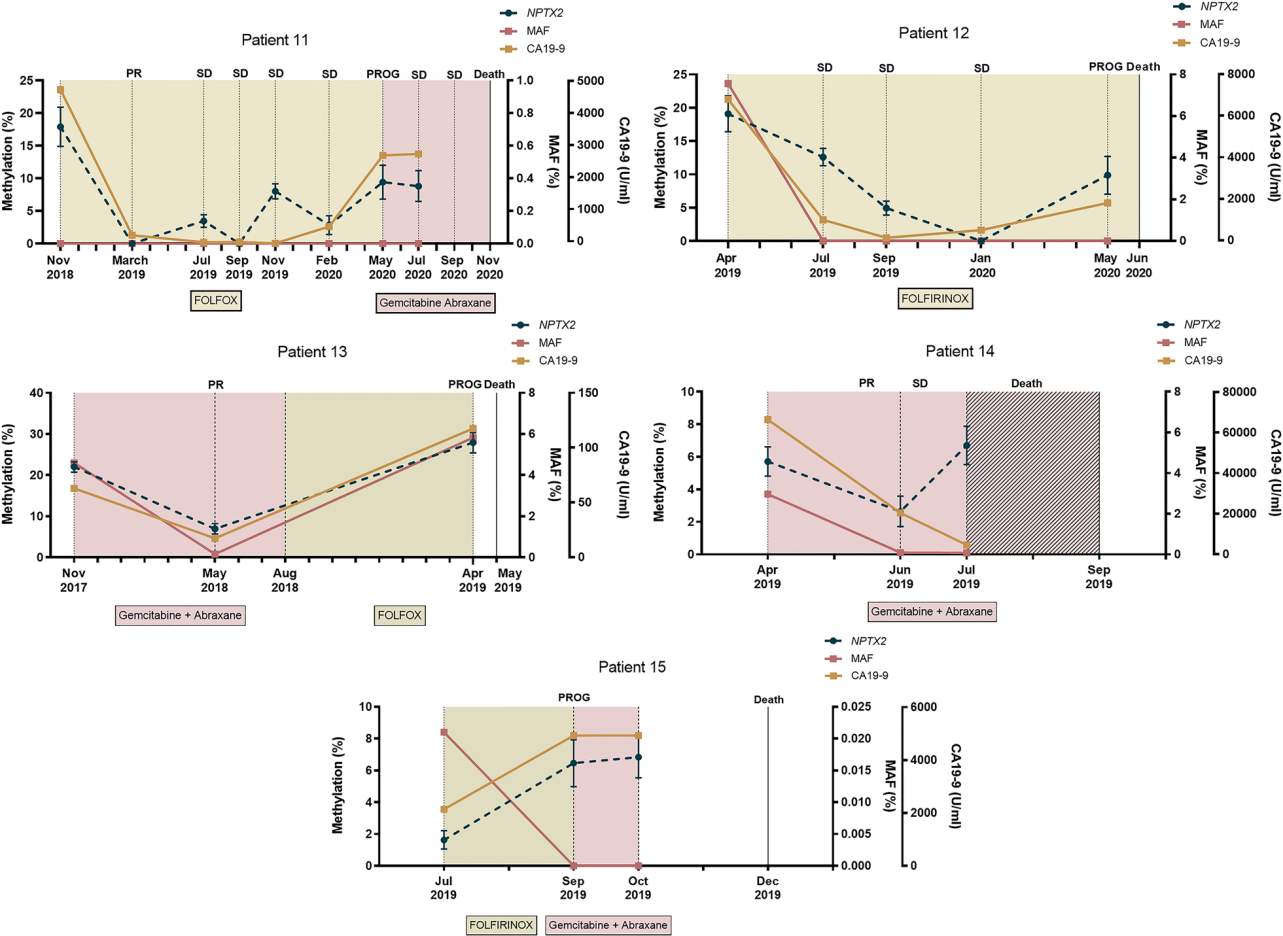
Circulating *NPTX2* methylation status for monitoring response to therapy and disease progression in mPDAC patients. The left *Y*-axis represents the percentage of *NPTX2* methylation in plasma. Right inner *Y*-axis represents the *RAS* mutant allele fraction (MAF, %) and right outer *Y*-axis represents CA19-9 (U/mL). The different treatments are indicated with different colors, and the shaded intervals indicate the lead time window of *NPTX2* in relation to disease progression. Bars indicate Poisson's error (95% CI)

**Fig. 6:**
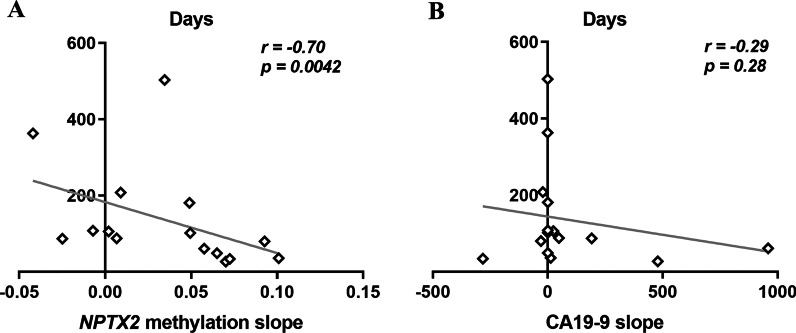
Circulating *NPTX2* methylation correlates with patient’s outcome and survival. Correlation analyses showed a significant inverse relationship between survival and circulating *NPTX2* methylation levels (**A**). But not with circulating CA19-9 levels (**B**)

There are many limitations in the use of CA19-9 as a reliable biomarker for the management of mPDAC patients. In this regard, we recently reported that dynamics of circulating *RAS* mutation may better correlate with mPDAC patients’ outcome and survival compared with standard CA19-9 marker [21]. Hence, here we next compared the utility of circulating *NPTX2* methylation with circulating CA19-9 and circulating *RAS* MAF for monitoring disease progression and response to therapy in 15 mPDAC patients. Overall, the dynamics of *NPTX2* methylation levels in cfDNA largely coincided with the dynamics of CA19-9 levels during the evolution of the disease for each patient (Fig. 5). However, whereas *NPTX2* methylation was detected in blood at baseline in 100% of the monitored patients, circulating *RAS* mutation or high CA19-9 levels (> 4515 U/mL) were found at diagnosis in 73.3% and 53.3% of the 15 patients analyzed respectively. After treatment initiation, *NPTX2* methylation was detected in 82.4% of the samples, whereas only 29.4% and 19.1% showed *RAS* mutation and high CA19-9 levels (> 4515 U/mL), respectively (Table 4). Moreover, in 27% of patients (4, 8, 12 and 15) no plasma *RAS* mutation was detected during their post-treatment follow-up, making this circulating biomarker less useful for patient monitoring. Of note, changes in circulating *NPTX2* methylation levels were substantiated by CT scans, with low or undetectable methylation levels at stable disease and high methylation levels at disease progression (Fig. 5). Importantly, in about half of the patients (2, 4, 5, 7, 8, 9 and 14) the disease progression detected by CT scan was anticipated by changes in circulating *NPTX2* methylation levels (Fig. 5). Moreover, in these patients *NPTX2* methylation changes occurred 100 ± 48 days ahead of disease progression. On the contrary, neither changes in circulating *RAS* MAF nor CA19-9 levels were so effective in foreseeing the evolution of the disease. Specifically, *NPTX2* methylation dynamics in plasmatic cfDNA correlated better with patient outcome and survival compared to the standard marker CA19-9. Thus, a significant correlation was found between increases in circulating *NPTX2* methylation levels and shorter survival periods (*r* = -0.70, *p* = 0.0042, Fig. 6A). On the contrary, no significant correlation with survival was observed for increases in CA19-9 levels (*r* = − 0.29, *p* = 0.28, Fig. 6B). Taken together, the above data support that dynamics of circulating *NPTX2* methylation predicts better than CA19-9 the evolution of disease in mPDAC patients.

**Table 4:** Characteristics of liquid biopsy samples

Variable	At diagnosis (%) *n* = 44	After treatment initiation (%) *n* = 51	Total samples (%) *n* = 95
CA19-9 (*n* = 90)			
> 4515 U/mL	22 (51.2%)	9 (19.1%)	34.4 (33.3%)
< 4515 U/mL	21 (48.8%)	38 (80.9%)	65.6 (66.7%)
cfDNA concentration			
> 31.8 ng/mL	22 (50%)	20 (39.2%)	42 (44.2%)
< 31.8 ng/mL	22 (50%)	31 (60.8%)	53 (55.8%)
*RAS* status in plasma			
*RAS* mutated	35 (79.5%)	15 (29.4%)	50 (52.6%)
*RAS* wild-type	9 (20.5%)	36 (70.6%)	45 (47.4%)
MAF			
> 2.01%	22 (50%)	2 (3.9%)	24 (25.3%)
< 2.01%	22 (50%)	49 (96.1%)	71 (74.7%)
*NPTX2*			
Methylated	35 (79.5%)	42 (82.4%)	77 (81.1%)
Unmethylated	9 (20.5%)	9 (17.6%)	18 (18.9%)
*NPTX2* methylation			
> 6.06%	26 (59.1%)	23 (45.1%)	49 (51.6%)
< 6.06%	18 (40.9%)	28 (54.9%)	46 (48.4%)

## Discussion

There is an urgent need of reliable biomarkers for the diagnosis, prognosis and patient monitoring in PDAC. CA19-9 is the commonly used circulating biomarker in the clinic for PDAC, having described the association between CA19-9 levels and survival in metastatic PDAC [9, 24]. However, in clinical practice there is no consensus on the interpretation of changes in CA19-9 levels during disease progression [10]. One of the leading alternatives is the use of liquid biopsy-based biomarkers, which allows to obtain real-time information on the evolution of the disease in a minimally invasive manner [25]. In this study we have explored the utility of liquid biopsy-based epigenetic biomarkers to monitor the response to treatment in patients with metastatic pancreatic ductal adenocarcinoma.

First, we have analyzed in cfDNA from eight mPDAC patients the methylation levels of five genes *BMP3*, *NPTX2*, *SPARC*, *TFPI2* and *SFRP1* known to be aberrantly methylated in pancreatic cancer. Of note, in all the eight patients, at least one of these genes were detected as methylated in cfDNA. Classified as tumor suppressors, these genes are epigenetically regulated. Specifically, BMP3 (Bone Morphogenetic Protein 3) acts as a direct regulator of genes involved in apoptosis and cell cycle arrest in pancreatic cancer [26]. SPARC (Secreted Protein Acidic and Cysteine Rich) is a glycoprotein involved in the inhibition of cell cycle progression or angiogenesis [26]. Low levels of *SPARC* expression have been described in ovarian, colorectal and pancreatic cancer, and *SPARC* promoter methylation has been proposed as an important factor in gastric carcinoma tumorigenesis [27]. TFPI2 (Tissue Factor Pathway Inhibitor 2) is a Kunitz-type serine proteinase inhibitor [28], which prevents the degradation of the extracellular matrix [29] and whose epigenetic inactivation contributes to the proliferation and invasiveness of tumors such as PDAC [30]. SFRP1 (Secreted Frizzled Related Protein 1) acts as a modulator of the Wnt pathway, and its hypermethylation has been associated with increased aggressiveness and decreased sensitivity to gemcitabine treatment in PDAC [31].

The *NPTX2* (Neuronal Pentraxin 2) gene codes for a protein of the neuronal pentraxin family, whose low expression implies an increase in tumor proliferation and metastasis [32]. Importantly, *NPTX2* methylation was the most prevalent in the cfDNA from mPDAC patients at diagnosis or after treatment, in agreement with previous studies where circulating *NPTX2* methylation was higher in PDAC patients with metastasis and advanced stage [20].

The decreased expression of *NPTX2* caused by promoter hypermethylation has a direct suppressive effect on the p53 signaling pathway, promoting processes such as proliferation and inhibiting apoptosis [32]. Besides, alteration of the p53 pathway has been previously pointed out as one of the main factors of progression in early stages of PDAC [33]. Our results, showing *NPTX2* methylation in 81.1% (77/95) of plasma samples, are consistent with those studies supporting that methylation of this gene must play a relevant role in the development and progression of PDAC [18, 20].

Most studies focused on the analysis of methylation levels of potential diagnostic and prognostic markers in cfDNA from pancreatic cancer patients employ the methylation status-specific PCR (MSP) technique, with quantitative [18, 20, 34–36] or non-quantitative determinations [37–40]. In this study we have used ddPCR, which is a more accurate and sensitive technique (lowering detection limits [22, 41, 42]), especially for samples with very low amounts of DNA [43, 44]. Moreover, we have increased the accuracy and reliability of determining *NPTX2* methylation levels by adding an amplification step before ddPCR.

Hypermethylation of *NPTX2* in pancreatic cancer tissue may constitute a molecular diagnostic marker [45], and *NPTX2* methylation levels in tumoral tissue have been associated with poor survival in PDAC patients [20]. In the present study, we show the association between circulating *NPTX2* methylation levels and other circulating biomarkers, including CA19-9 levels, cfDNA concentration, and circulating *RAS* mutational status, which has recently been described as a prognostic biomarker in mPDAC [21]. Moreover, our survival analyses demonstrated the value of basal circulating NPTX2 methylation for risk-stratification of mPDAC patients. Therefore, our results support circulating *NPTX2* methylation as a relevant epigenetic biomarker that may constitute a valuable prognostic tool in the management of mPDAC patients.

Very few studies have described the analysis of methylated cfDNA for monitoring the evolution of disease in cancer patients [46, 47]. Moreover, to our knowledge, no previous studies have explored the utility of cfDNA methylation levels for monitoring the progression and response to therapy in pancreatic cancer. In our study we demonstrate that the dynamics of *NPTX2* methylation is closely associated with the clinical course of the disease. Similar trends were frequently observed for *NPTX2* methylation and CA19-9 levels, although cfDNA methylation was a more accurate indicator of the patient´s outcome.

Hence, elevated circulating *NPTX2* methylation levels were found at diagnosis or when the disease progressed and were maintained or decreased when partial response or stable disease outcome occurred. Moreover, in several patients, *NPTX2* methylation dynamics were able to anticipate the disease progression detected by CT scans and CA19-9 tests. Therefore, our results show that circulating *NPTX2* methylation levels during disease monitoring offers to clinicians a wider window opportunity in which the treatment regimens could be modified.

Lastly, future research is warranted for the validation of our findings in separate patient and control cohorts, encompassing diverse disease stages, utilizing alternative techniques (such as pyrosequencing, for instance), and analyzing other markers that have been reported in the literature as promising diagnostic and prognostic targets, including but not limited to *BNC1* [19, 35–37, 48–50], *SEPT9* [48, 50], *ADAMTS1*, *HOXA1*, *PCDH10*, *SEMA5A*, or *SPSB4* [34].

## Conclusions

In this study we evaluated liquid biopsy-based epigenetic biomarkers as prognostic and monitoring disease tools in metastatic PDAC patients. Our results show that higher circulating *NPTX2* methylation levels at diagnosis were associated with poor prognosis and efficiently stratified patients for prediction of OS. Dynamics of *NPTX2* methylation correlated with disease progression and treatment response, predicting better than CA19-9 the evolution of disease in mPDAC patients. Notably, *NPTX2* methylation dynamics were able to anticipate the disease progression detected by CT scans and CA19-9. Our study supports circulating *NPTX2* methylation levels as a promising liquid biopsy-based clinical tool for the non-invasive prognosis, monitoring disease evolution and response to treatment in PDAC patients.

## Supplementary Information

**Additional file 1: Table S1.** Primers and probes for methylation analysis of different genes in cfDNA. **Table S2.** Comparison of *NPTX2* methylation levels and Poisson’s error in patient samples at diagnosis and after initiation of treatment with and without pre-ddPCR amplification.

**Additional file 2: Figure S1.** Comparative analysis of methylation data of *BMP3*, *NPTX2*, *SFRP1*, *SPARC* and *TFPI2* genes in normal and tumor tissue from pancreatic adenocarcinoma patients in TCGA database. Box-plot graphs show the aggregated mean *β*-values of the island CGs available in TCGA for each gene. All 5 genes are significantly hypermethylated in tumor (*n* = 184) compared with normal (*n* = 10) tissue samples.

**Additional file 3: Figure S2.** ddPCR quality control parameters for an accurate quantification of *NPTX2* methylation. The results of patients 14 and 15 throughout their monitoring (-1, -2 and -3) are shown. All the preamplified cfDNA samples were submitted to a ddPCR reaction whereas the quality control parameters were evaluated. **A**, **B** Representative 1D amplitude plot showing the droplets. The threshold separating positive and negative droplets was set at (**A**) 4000 for methylated copies (FAM probe) and (**B**) 5000 for unmethylated copies (SUN probe). **C** Bi-dimensional plots showing unmethylated *NPTX2* positive droplet clusters (blue dots), methylated *NPTX2* positive droplet clusters (green dots), negative droplet clusters (dark grey dots) and double positive droplets (orange dots). **D** All samples had more than 10,000 total droplets detected and a minimum number of positive droplets ≥ 3 was considered to call a positive sample for methylated *NPTX2* detection. **E** Fractional abundance (methylated copies (FAM)/(methylated copies (FAM) + unmethylated copies (SUN) × 100).

**Additional file 4: Figure S3.** ddPCR results for *NPTX2* methylation analysis in healthy individuals and methylation control samples. **A**, **B** 1D amplitude plots showing the positive droplets for methylated and unmethylated state of *NPTX2* in two healthy individuals (h) and commercial controls (c): negative (0% methylated), positive (100% methylated) and 50% (50% methylated). The threshold separating positive and negative droplets was set at A 4000 for methylated copies (FAM probe) and **B** 5000 for unmethylated copies (SUN probe). **C** Bar representation showing the total and positive droplet count for methylated and unmethylated state of *NPTX2*.

**Additional file 5: Figure S4.** ddPCR results for *BMP3*, *SPARC*, *SFRP1* and *TFPI2* methylation analysis in healthy individuals and methylation control samples. **A**, **B** 1D amplitude plots showing the positive droplets for methylated state of each gene (**A**) and for Actin-1 probe (**B**) in commercial 100% methylated control (c100) and healthy individual (h). The threshold separating positive and negative droplets was set at **A** 3000 for *BMP3*, *SPARC* and *SFRP1* methylated copies (FAM probe) and 4000 for *TFPI2* methylated copies (FAM probe); and **B** 3000 for *Actin1* copies (SUN probe). **C** Bar graph showing the total and positive droplet counts for methylated state of *BMP3*, *SPARC*, *SFRP1* and *TFPI2* and for *Actin1* molecules.

## Data Availability

Not applicable.

## Abbreviations

CT: Computed tomography
cfDNA: Cell-free DNA
ddPCR: Digital droplet PCR
MAF: Mutant allele fraction
mPDAC: Metastatic pancreatic ductal adenocarcinoma
OS: Overall survival
PFS: Progression-free survival
