# Supplementary material for: Circulating NPTX2 methylation as a non-invasive biomarker for prognosis and monitoring of metastatic pancreatic cancer
Source: Clin Epigenetics. 2023 Jul 22;15:118. doi: 10.1186/s13148-023-01535-4 (PMC10362605; doi:10.1186/s13148-023-01535-4)
Supplement: Supplementary file 1 — Additional file 1: Table S1. Primers and probes for methylation analysis of different genes in cfDNA. Table S2. Comparison of NPTX2 methylation levels and Poisson’s error in patient samples at diagnosis and after initiation of treatment with and without pre-ddPCR amplification. [file 13148_2023_1535_MOESM1_ESM.docx]

**Supplementary Table 1. Primers and probes for methylation analysis of different genes in cfDNA.**

| **Target** | **Name** | **Sequence 5’- 3’** |
| --- | --- | --- |
| *BMP3* | Forward M^(1)^ | TGGAGACGGCGTTCGTA |
|  | Reverse M^(1)^ | CCACTTACTACGCTAACCCAAC |
|  | Probe M^(1)^ | /56-FAM/CG GGT GAG G/ZEN/T TCG CGT AGT TGT T/3IABkFQ/ |
| *NPTX2* | Forward M^(1)^ | GGTTGTGAGACGGTAGGAGAT |
|  | Reverse M^(1,2)^ | CGCGCTCAACTCGACATAAA |
|  | Probe M^(1,2)^ | /56-FAM/AC GCC TTC G/ZEN/C TAC TAA CAA TAA CCG C/3IABkFQ/ |
|  | Forward Y^(2)^ | GGTTGTGAGAYGGTAGGAGAT |
|  | Reverse U^(2)^ | CCACACTCAACTCAACATAAAC |
|  | Probe U^(2)^ | /5SUN/CACCTTCACTACTAACAATAACCACAC |
| *SPARC* | Forward M^(1)^ | TTTCGCGGTTTTTTAGATTGTTC |
|  | Reverse M^(1)^ | AACGACGTAAACGAAAATATCG |
|  | Probe M^(1)^ | /56-FAM/AG CGC GTT T/ZEN/T GTT TGT CGT TTG TTT G/3IABkFQ/ |
| *SFRP1* | Forward M^(1)^ | GTGGTAACGAGTGCGGTT |
|  | Reverse M^(1)^ | AAACGCGAAACGACGAAC |
|  | Probe M^(1)^ | /56-FAM/TT TCG TCG G/ZEN/G AGT TGA TTG GTT GC/3IABkFQ/ |
| *TFPI2* | Forward M^(1)^ | TTTGGAGTAGAAAGTCGCGTAT |
|  | Reverse M^(1)^ | CCCAATACAACCTCCGTCAA |
|  | Probe M^(1)^ | /56-FAM/TT TGT TTA GCG GGT CGT TCG ATT T/3IABkFQ/ |
| *ACTB* | Forward M^(1)^ | GAAAGATATAAGGTTAGGGATAGGG |
|  | Reverse M^(1)^ | CCAACACACAATAACAAACACA |
|  | Probe M^(1)^ | /5SUN/AC TAA ACC T/ZEN/C CTC CAT CAC CAC CC/3IABkFQ/ |

^(1)^For analysis using β-Actin as reference gene for normalization.

^(2)^For analysis using methylated and unmethylated molecules of the gene as a normalizing factor.

**Supplementary Table 2. Comparison of *NPTX2* methylation levels and Poisson's error in patient samples at diagnosis and after initiation of treatment with and without pre-ddPCR amplification.**

|  |  | ***NPTX2* Methylation (%) ± Poisson Error** | |
| --- | --- | --- | --- |
| **Patient** | **Monitoring sample** | **without preamplification** | **with preamplification** |
| **1** | 1 | 3.84 ± 4.17 | 0.961 ± 0.439 |
|  | 2 | 3.92 ± 2.85 | 3.15 ± 0.73 |
|  | 3 | 5.25 ± 2.62 | 0.341 ± 0.253 |
|  | 4 | 10.3 ± 4.1 | 0.424 ± 0.309 |
|  | 5 | 10 ± 7 | 6.95 ± 1.63 |
| **2** | 1 | 0 | 1.78 ± 0.73 |
|  | 2 | 2.56 ± 2.8 | 0 |
|  | 3 | 1.28 ± 1.41 | 0.275 ± 0.25 |
|  | 4 | 7.56 ± 3.34 | 1.64 ± 0.58 |
|  | 5 | 3.7 ± 2.15 | 0.112 ± 0.123 |
|  | 6 | 3.63 ± 2.65 | 2.43 ± 0.65 |
|  | 7 | 0 | 2.73 ± 0.81 |
| **3** | 1 | 4.64 ± 3.37 | 5.89 ± 1.01 |
|  | 2 | 0 | 1.67 ± 0.6 |
|  | 3 | 0 | 0.353 ± 0.39 |
|  | 4 | 3.57 ± 3.87 | 7.21 ± 1.18 |
| **4** | 1 | 0 | 6.24 ± 0.96 |
|  | 2 | 0 | 18.2 ± 2.7 |
| **5** | 1 | 6.06 ± 4.34 | 18.9 ± 2.3 |
|  | 2 | 0 | 0 |
|  | 3 | 6.25 ± 6.65 | 0.447 ± 0.495 |
| **6** | 1 | 32.5 ± 7.2 | 51.2 ± 2.6 |
|  | 2 | 4.16 ± 3.03 | 8.86 ± 1.34 |
|  | 3 | 7.89 ± 4.51 | 5.43 ± 1.33 |
|  | 4 | 32 ± 9.4 | 14.7 ± 3.3 |
| **7** | 1 | 12.5 ± 8.6 | 0.947 ± 0.52 |
|  | 2 | 16.7 ± 11.2 | 2.13 ± 1.26 |
|  | 3 | 0 | 7.7 ± 2.24 |
|  | 4 | 0 | 12.2 ± 2.6 |
| **8** | 1 | 12.5 ± 6 | 20.9 ± 6.2 |
|  | 2 | 0 | 0 |
|  | 3 | 2.63 ± 2.87 | 3.39 ± 0.6 |
| **9** | 1 | 16 ± 7.5 | 5.86 ± 2.35 |
|  | 2 | 8 ± 5.7 | 9.27 ± 1.93 |
|  | 3 | 8.33 ± 8.77 | 0 |
|  | 4 | 6.66 ± 7.14 | 0 |
|  | 5 | 9.52 ± 6.68 | 0 |
|  | 6 | 8.57 ± 4.83 | 11 ± 1.9 |
|  | 7 | 21 ± 6.7 | 25.5 ± 3.6 |
|  | 8 | 9.08 ± 5.12 | 21.1 ± 2.2 |
| **10** | 1 | 0 | 3.34 ± 1.5 |
|  | 2 | 2.77 ± 3.03 | 6.45 ± 1.48 |
|  | 3 | 4.25 ± 3.09 | 6.62 ± 1.56 |
|  | 4 | 0 | 4.76 ± 5.13 |
| **11** | 1 | 5 ± 5.4 | 17.9 ± 3 |
|  | 2 | 0 | 0 |
|  | 3 | 3.7 ± 2.7 | 3.47 ± 0.95 |
|  | 4 | 11.4 ± 4.8 | 0 |
|  | 5 | 4.76 ± 3.44 | 8.03 ± 1.16 |
|  | 6 | 5.26 ± 5.64 | 2.84 ± 1.44 |
|  | 7 | 4 ± 4.32 | 9.42 ± 2.58 |
|  | 8 | 0 | 8.81 ± 2.39 |
| **12** | 1 | 17.8 ± 7.4 | 19.1 ± 2.7 |
|  | 2 | 11.3 ± 3.7 | 12.6 ± 1.3 |
|  | 3 | 1.75 ± 1.92 | 4.94 ± 1.05 |
|  | 4 | 0 | 0 |
|  | 5 | 11.8 ± 8.1 | 9.89 ± 2.81 |
| **13** | 1 | 26.2 ± 4.3 | 22 ± 1.2 |
|  | 2 | 0 | 6.95 ± 1.27 |
|  | 3 | 26.1 ± 9.2 | 27.9 ± 2.4 |
| **14** | 1 | 7.49 ± 4.31 | 5.71 ± 0.9 |
|  | 2 | 6.25 ± 6.65 | 2.64 ± 0.94 |
|  | 3 | 5.26 ± 5.64 | 6.7 ± 1.18 |
| **15** | 1 | 2.85 ± 3.12 | 1.63 ± 0.58 |
|  | 2 | 4.16 ± 3.03 | 6.46 ± 1.47 |
|  | 3 | 5 ± 5.4 | 6.84 ± 1.29 |
