# Supplementary figures and images for: Circulating NPTX2 methylation as a non-invasive biomarker for prognosis and monitoring of metastatic pancreatic cancer
Source: Clin Epigenetics. 2023 Jul 22;15:118. doi: 10.1186/s13148-023-01535-4 (PMC10362605; doi:10.1186/s13148-023-01535-4)

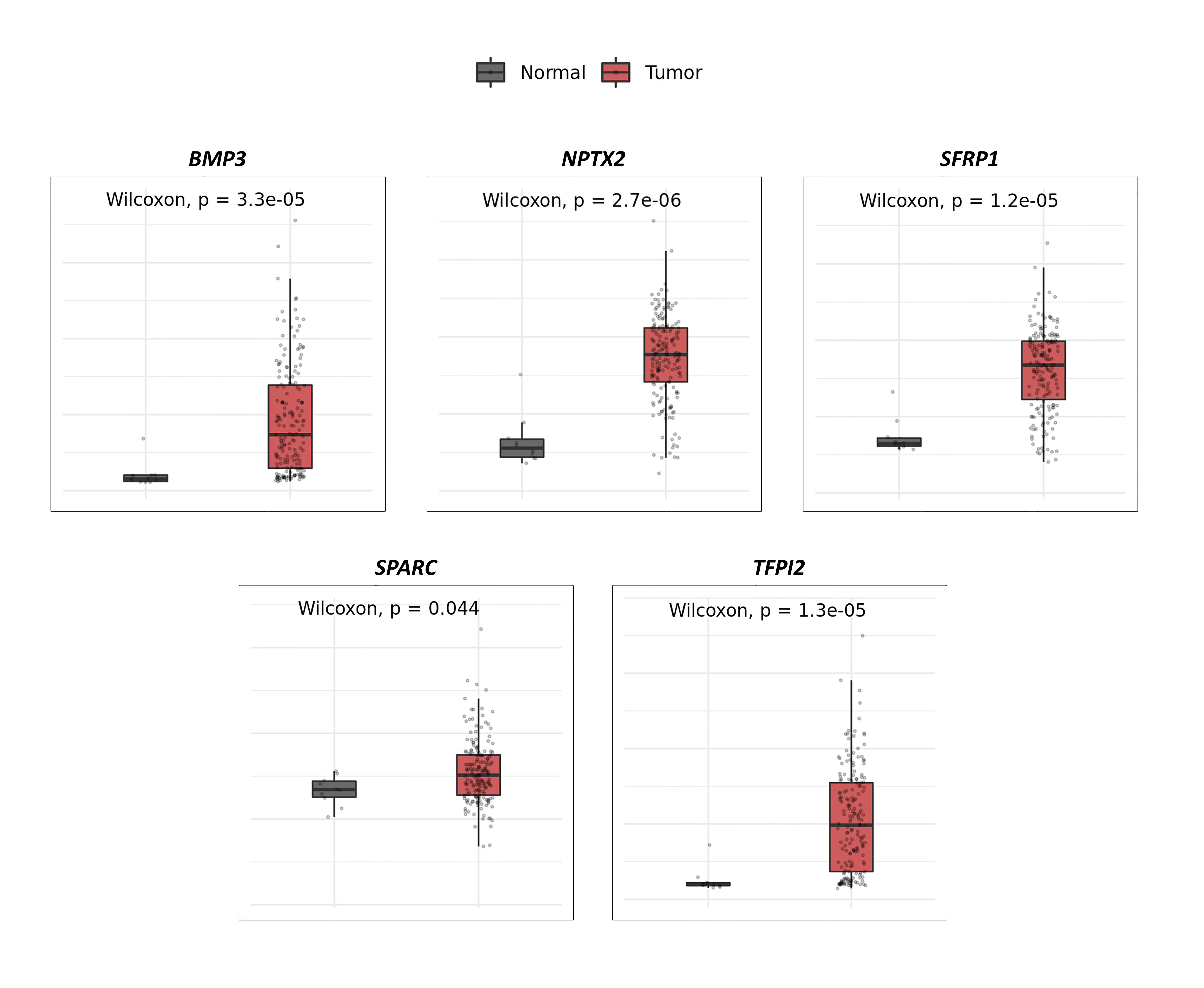

Supplement: Supplementary file 2 — Additional file 2: Figure S1. Comparative analysis of methylation data of BMP3, NPTX2, SFRP1, SPARC and TFPI2 genes in normal and tumor tissue from pancreatic adenocarcinoma patients in TCGA database. Box-plot graphs show the aggregated mean β-values of the island CGs available in TCGA for each gene. All 5 genes are significantly hypermethylated in tumor (n = 184) compared with normal (n = 10) tissue samples. [file 13148_2023_1535_MOESM2_ESM.jpg]

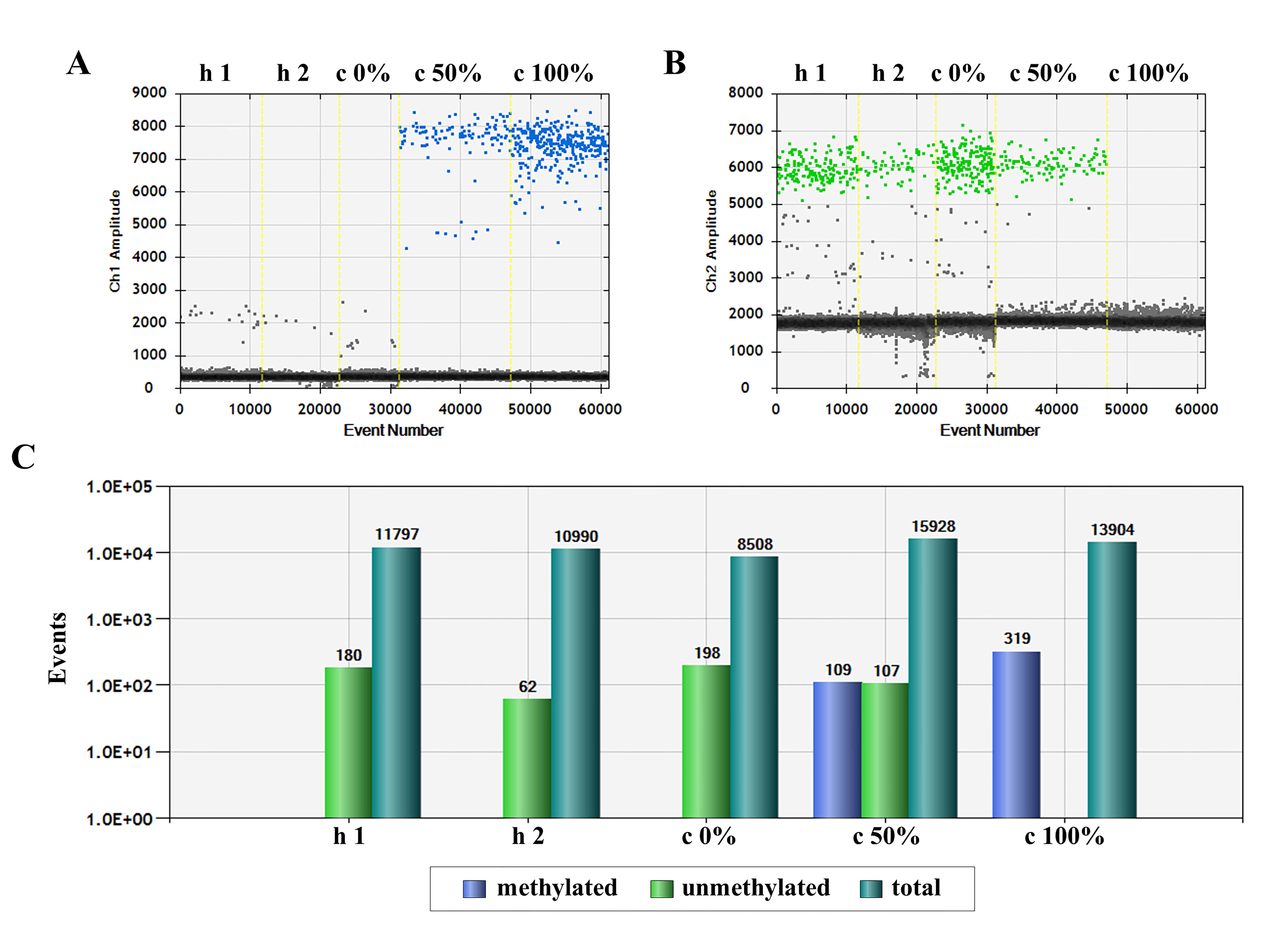

Supplement: Supplementary file 4 — Additional file 4: Figure S3. ddPCR results for NPTX2 methylation analysis in healthy individuals and methylation control samples. A, B 1D amplitude plots showing the positive droplets for methylated and unmethylated state of NPTX2 in two healthy individuals (h) and commercial controls (c): negative (0% methylated), positive (100% methylated) and 50% (50% methylated). The threshold separating positive and negative droplets was set at A 4000 for methylated copies (FAM probe) and B 5000 for unmethylated copies (SUN probe). C Bar representation showing the total and positive droplet count for methylated and unmethylated state of NPTX2. [file 13148_2023_1535_MOESM4_ESM.jpg]

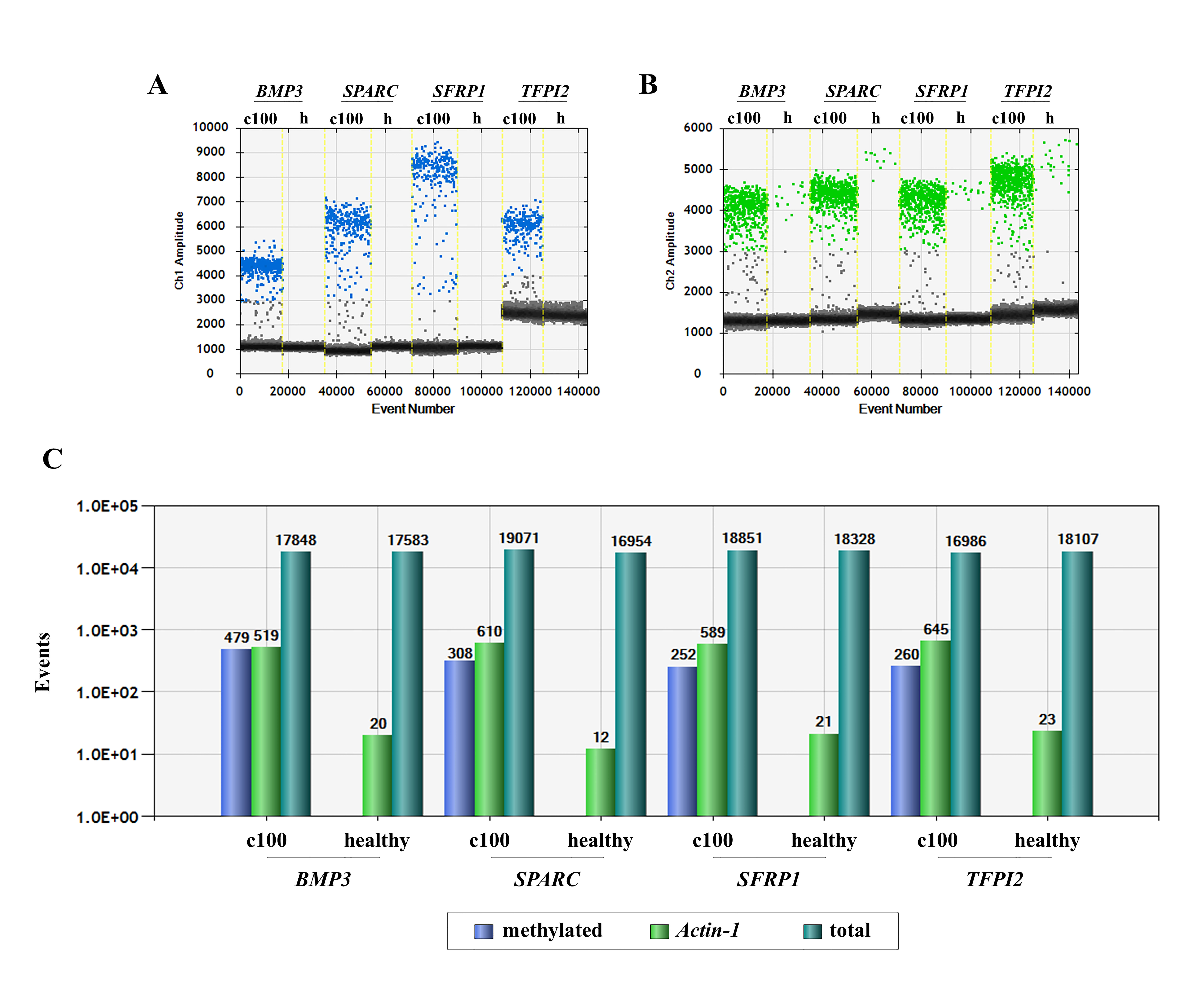

Supplement: Supplementary file 5 — Additional file 5: Figure S4. ddPCR results for BMP3, SPARC, SFRP1 and TFPI2 methylation analysis in healthy individuals and methylation control samples. A, B 1D amplitude plots showing the positive droplets for methylated state of each gene (A) and for Actin-1 probe (B) in commercial 100% methylated control (c100) and healthy individual (h). The threshold separating positive and negative droplets was set at A 3000 for BMP3, SPARC and SFRP1 methylated copies (FAM probe) and 4000 for TFPI2 methylated copies (FAM probe); and B 3000 for Actin1 copies (SUN probe). C Bar graph showing the total and positive droplet counts for methylated state of BMP3, SPARC, SFRP1 and TFPI2 and for Actin1 molecules. [file 13148_2023_1535_MOESM5_ESM.jpg]
